# Thermal degradation of aqueous 2-aminoethylethanolamine in CO_2_ capture; identification of degradation products, reaction mechanisms and computational studies

**DOI:** 10.1186/s13065-016-0231-7

**Published:** 2017-01-24

**Authors:** Idris Mohamed Saeed, Vannajan Sanghiran Lee, Shaukat Ali Mazari, B. Si Ali, Wan Jeffrey Basirun, Anam Asghar, Lubna Ghalib, Badrul Mohamed Jan

**Affiliations:** 1 Department of Chemical Engineering, Faculty of Engineering, University of Malaya, 50603 Kuala Lumpur, Malaysia; 2 Department of Chemistry, Faculty of Science, University of Malaya, 50603 Kuala Lumpur, Malaysia; 3 Department of Chemical Engineering, Dawood University of Engineering and Technology, Karachi, 74800 Pakistan

**Keywords:** 2-aminoethylethanolamine (AEEA), CO_2_ capture, Thermal degradation, Mechanism, Computational study

## Abstract

Amine degradation is the main significant problems in amine-based post-combustion CO_2_ capture, causes foaming, increase in viscosity, corrosion, fouling as well as environmental issues. Therefore it is very important to develop the most efficient solvent with high thermal and chemical stability. This study investigated thermal degradation of aqueous 30% 2-aminoethylethanolamine (AEEA) using 316 stainless steel cylinders in the presence and absence of CO_2_ for 4 weeks. The degradation products were identified by gas chromatography mass spectrometry (GC/MS) and liquid chromatography-time-of-flight-mass spectrometry (LC-QTOF/MS). The results showed AEEA is stable in the absence of CO_2_, while in the presence of CO_2_ AEEA showed to be very unstable and numbers of degradation products were identified. 1-(2-Hydroxyethyl)-2-imidazolidinone (HEIA) was the most abundance degradation product. A possible mechanism for the thermal degradation of AEEA has been developed to explain the formation of degradation products. In addition, the reaction energy of formation of the most abundance degradation product HEIA was calculated using quantum mechanical calculation.

## Background

Post-combustion CO_2_ capture is a topic of the environmental and climatic mitigation of carbon based energy system. Several studies have surveyed the environmental and climatic mitigation of carbon based energy system [1], and the impacts of lower-pollution energy system transition [2], including natural gas others [3, 4]. One of obvious conclusions is that without carbon capture and storage, carbon based energy system could not avoid the additional global warming. Post-combustion CO_2_ capture would reduce the pollutants and carbon emission, and increase environmental and climatic health. Post-combustion based on amine CO_2_ capture is the most dominant technology used for CO_2_ capture. This technique uses different aqueous alkanolamines to absorb CO_2_ gas from flue gas stream. This technology has several advantages such as good reactivity, high capacity and low cost. Moreover, the alkanolamines can be recovered after the completion of the whole process [5, 6].

However, alkanolamines also undergo irreversible reaction with acid gases to produce undesired compounds. Alkanolamines suffers from thermal and oxidative degradation. Thermal degradation occurs due to the high temperature in the stripper, and may also occur in the cross heat exchanger and the reclaimer, depending on the configuration [7, 8]. Degradation of amines is undesirable for amine-based CO_2_ capture as this causes growing economic burden and may cause operating problems like fouling, corrosion and foaming [9–11]. Amine degradation is one of the major issues associated with amine based post combustion carbon capture (PCC). The degradation products from the process causes foaming, increased viscosity, high corrosion of equipment and fouling [12, 13]. Furthermore, emissions and disposal of degradation products cause environmental and health issues. These contribute to economic glitches, which requires urgent panacea.

In recent years, new solvent such as 2-Aminoethylethanolamine (AEEA) has been utilized as an absorbent for CO_2_ from post-combustion exhaust gases [14, 15]. AEEA is a diamine, which contains two nitrogen atoms that can absorb CO_2_ and one OH group which increases the solubility in aqueous solution. AEEA exhibit better performance than other industrial amine such as N-methylethanolamine (MEA) [15], due to the higher solubility, lower vapor pressure, higher absorption capacity, greater heat absorption and lower desorption energy [14–18].

There is a lack of data regarding to the thermal degradation investigation of AEEA-based CO_2_ scrubbing system, and the degradation product formation pathways and that require further research. In this article, the thermal degradation of 30% AEEA is presented in detail. Identification, reaction mechanism, computational chemistry studies of the degradation products are proposed and discussed.

## Experimental methods

### Materials

All chemicals such as 2-aminoethylethanolamine (AEEA) (≥98%), barium chloride (BaCl_2_) and standards solutions of hydrochloric acid (HCl), sodium hydroxide (NaOH) and sulfuric acid (H_2_SO_4_) were procured from Merck (Malaysia). Carbon dioxide (99%) and N_2_ (≥99.99%) gases were procured from a Linder (Malaysia). All chemicals were used as purchased without further purification.

### Sample preparation and CO_2_ loading experiments

**Fig. 1:**
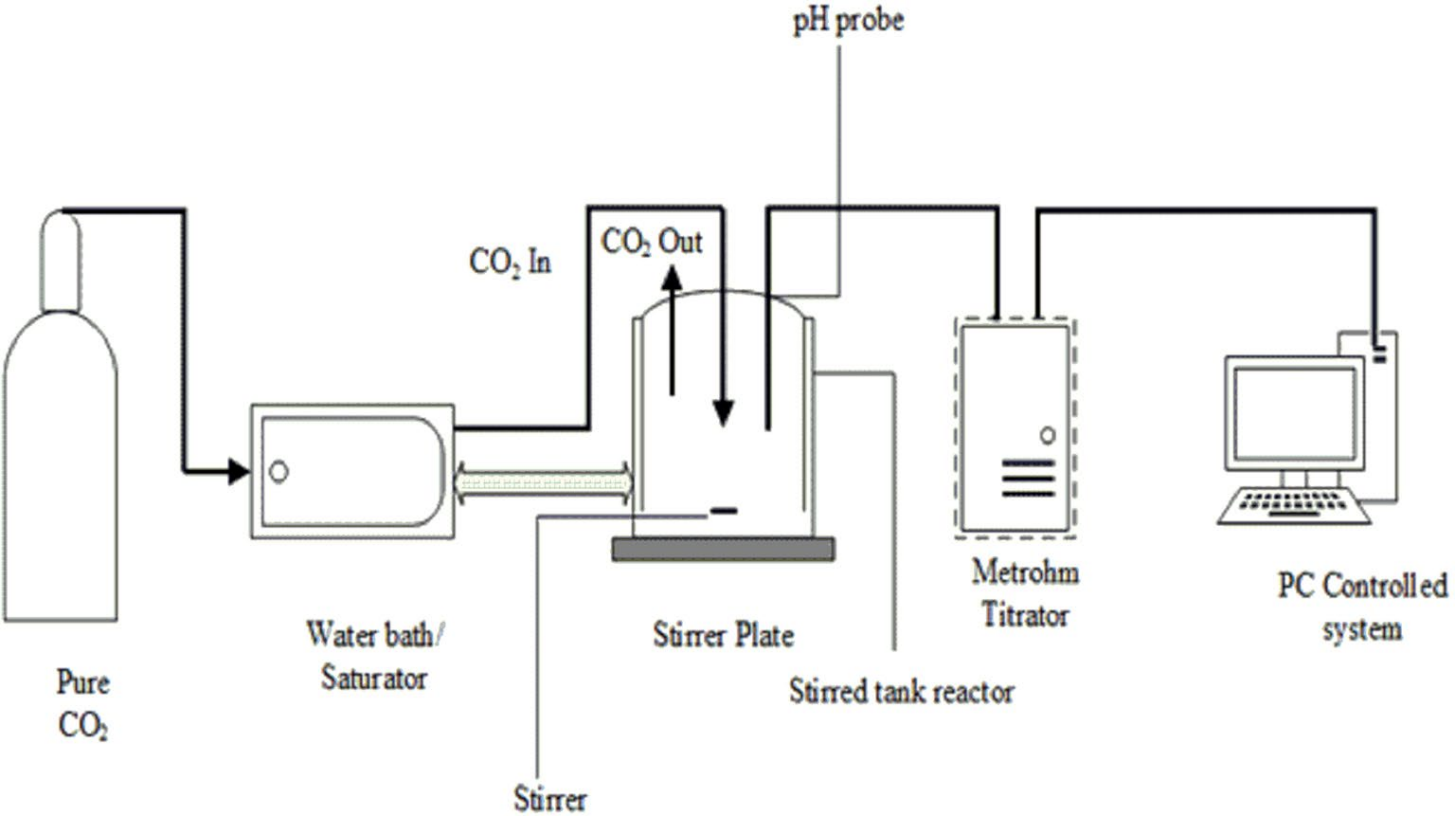
Schematic diagram of CO_2_ loading setup for amine saturation method

An aqueous AEEA solution was prepared in weight percent with concentrations of 30 wt% and diluted using di-ionized water. In the CO_2_ loading experiment, the reactor was equipped with a magnetic stirrer, and a pH meter linked to data acquisition system. A pH Probe linked to Metrohm was used to monitor the solution pH versus time. Figure 1 provides a description of the experimental setup. The reaction started by introducing a volume of 100 ml of 30% wt of aqueous AEEA into a double jacket reactor. The solution was then purged with nitrogen gas for 5 min to remove any possible dissolved oxygen. The CO_2_ gas was introduced into the reactor until it became saturated with CO_2_ with pH 7. Then samples were taken and transferred into the cylinders and CO_2_ loading was verified by titration [19]. In CO_2_ loading determination, two samples of nearly 0.5 g were withdrawn form reactor, and transferred into a mixture of 50 ml NaOH (0.1 M) and 25 ml of BaCl_2_ (0.5 M) in a 250 ml Erlenmeyer flask. The reaction of BaCl_2_ and NaOH with CO_2_ result in the formation of white precipitates of BaCO_3_. Samples were heated, then cooled and filtered using 0.45 µm pore size silicon filter paper. The white crystals were washed with 50 ml deionized water, and then 0.1 M HCl was added until all crystals dissolved. Acidified samples were then titrated with 0.1 M NaOH. All the titration tasks were performed using 785 DMP Titrino auto-titrator installed with Tiamo 1.3–45.

### Thermal degradation experiments

The thermal degradation experiment was performed in a metal cylinder (5 in. length and ½ in.outer diameter) made from 316 stainless steel and equipped with Swagelok end-caps. The method used was similar to Davis et al. [20]. 8 ml of sample with and without CO_2_ were introduced directly into the cylinder, placed in a Memmert 600 oven and heated at 135 °C, above the stripper temperature (to accelerate the reaction). Experiments were conducted at high temperature 135 °C as intention was to accelerate the thermal degradation to produce highly degraded samples within a reasonable timeframe.

The cylinders were periodically removed from the oven (once per week) during the whole 4 weeks. Any suspected leakage was checked by the weight differences of before and after the experiments. After the cylinders were cooled to room temperature, the samples were transferred to vials and kept refrigerated at 5 °C to quench the reaction and finally subjected to further analysis.

### Analytical methods

#### Gas chromatography–mass spectrometry (GC–MS)

The GC–MS instrument (model 6890 N/5973 N) was from Shimadzu coupled with mass spectrometer (MS) using Shimadzu GCMS-QP2010, with Ultra autosampler AOC 20I+S. The separation of amines and degradation products were performed in RTX ^®^-5MS column using the conditions shown in Table 1. Each sample was diluted using methanol mixture in the ratio of 1:50 prior to the analysis to avoid contamination of the system and to provide a higher sensitivity. The analysis was performed for 30 min to ensure the elution of heavy degradation compounds.

**Table 1:** GC–MS parameters for identifications of degradation products

Column	RTX®-5MS
Length (m)	30.00
Internal diameter (µm)	0.25 mm
Thickness (µm)	0.25 µm
Initial temp. (°C)	70
Initial hold time (min)	1
Oven ramp (1) (°C min^–1^)	5
Oven ramp (2) (°C min^–1^)	5
Final temp. (°C)	240
Final hold time (min)	10
Injector temp. (°C)	300
Flow rate (constant) (ml min^−1^)	1
Carrier gas	He

#### Liquid chromatography-time-of-flight-mass spectrometry (LC-QTOF-MS)

The analyses of the degraded samples were carried out using an Agilent 1260 infinity liquid chromatography coupled with 6224 time-of-flight (TOF) MS. The molecules were converted to ions by an electrospray ionization source (ESI). The column used was Zorbax Eclipse Plus (2.1 × 100 mm) and the amount of injected volume was 20 µl. The eluent was 0.10% formic acid in water (1) and methanol (2). A gradient profile is described in Table 2. The flow rate was set at 0.200 ml/min. The method developed by Huang et al. [21] was used for the detection of the degradation products.

**Table 2:** Gradient profile for the mobile phase ratio in this experiment

Time (min)	Mobile phase ratio	
	Formic acid (0.10%)	Methanol (99.9%)
6	98	2
2	20	80
8–14	98	2

## Computational details

All the transition state structures and reactants were fully optimized, in the gas phase at 298.15K at B3LYP/6-311++G(d, p) level of theory using the Gaussian09 [22] and GaussView visualization program [23]. The transition state calculations of the proposed mechanisms were carried out. Synchronous transition methods were used to find a transition state (TS) under D mol^3^ module in Material Studio 4.4 for the structure optimization and reaction path calculations. All calculations were performed using the density functional theory (DFT) with local density approximation (LDA) of local functional PWC [24], with effective core potential treatment with the DN basis set. The reaction paths were obtained using the linear synchronous transit (LST) and optimization calculation performs a single interpolation to a maximum energy, followed by the quadratic synchronous transit (QST) method, for an energy maximum with constrained minimizations in order to refine the transition state to a high degree [25]. Another conjugate gradient minimization was performed at each point. The cycle was repeated until a stationary point was located or the number of allowed QST steps was exhausted. After the initial paths were converged, the highest energy points were optimized to the closest transition state (TS). Following the TS optimization, the minimum energy path (MEP) between the critical points were calculated with the nudged elastic band (NEB), to ensure continuity of the path and projection of the force, so that the system converges to the MEP. The TS were checked at the B3LYP/6-311++G (d, p) level by evaluating the vibrational frequencies. The optimized geometries obtained were characterized as stationary points on the potential energy surface (PES) and the transition states were characterized by only one imaginary frequency, which is confirmed to represent the most accurate reaction coordinate. The computational method used in this study is similar to Lee et al. [26].

## Results and discussions

The investigation of formation of thermal degradation products in AEEA system was conducted in three different conditions; thermal degradation in the absence of CO_2_ (AEEA/H_2_O), thermal degradation in the presence of CO_2_ (AEEA/H_2_O/CO_2_) and quantum mechanical calculations of the formation of the main degradation product (HEIA). In the AEEA/H_2_O system, the aqueous amine solution was heated at 135 °C for 4 weeks. In the AEEA/H_2_O/CO_2_ system, the amine solution was first loaded with CO_2_ (α = 0.80 mol CO_2_/mol of amine) and then heated to 135 °C for 4 weeks. At the end of each experiment, the liquid phase analysis was carried out by using GC–MS and LC-QTOF-MS to identify the degradation products.

### Identification of degradation products

**Fig. 2:**
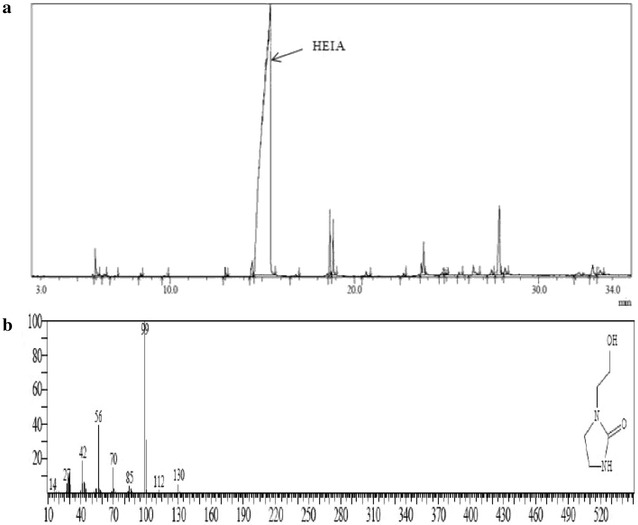
Gas chromatogram (**a**) and mass spectrum of HEIA (**b**) of aqueous AEEA solution After 4 weeks of the experiment using 30 wt% AEEA at temperature 135 °C with 0.80 moleCO_2_/mol of amine

The identification of amine degradation products were performed by GC–MS and LC-MS-QTOF techniques which are listed in Table 2. No degradation products were identified during the thermal degradation of AEEA in the absence of CO_2_. However, 27 degradation products were detected during the thermal degradation of AEEA in the presence of CO_2_. 1-(2-Hydroxyethyl)-2-imidazolidinone (HEIA) was the most abundant degradation product in the system, Fig. 2 represents the GC chromatogram of a sample of the thermal degradation of AEEA and mass spectrum of HEIA after 4 weeks. Low molecular weight volatile compounds such as ammonia are other degradation products which were likely to be formed in the process. However, the analytical methods employed in this work could not facilitate the detection. In this study, heat stable salts (HSS) such as formate, acetate were identified in this study in agreement with literature [27, 28]. However, the presence of HSS due to presence of oxygen in head space inside the cylinder as by-products of CO_2_ reduction that formed during degradation [28]. However, those products couldn’t be identified due to the limitations of our analytical techniques (Table 3).

**Table 3:** Compounds identified by the present study by using GC-MS and LC-MS-QTOF in AEEA/CO_2_/H_2_O system at 135 °C

Compound	Abb.	MW (g/mol)	Structures	Analytical technique
Diethanolamine	DEA	105		GC–MS LC-MS-QTOF
1-Piperazineethanol	HEP	126		GC–MS LC-MS-QTOF
1,4-Bis(2-hydroxyethyl)piperazine	BHEP	130		GC–MS LC-MS-QTOF
2-Imidazolidinone	HEI	86		GC–MS
1-(2-Hydroxyethyl)-2-imidazolidinone	HEIA	130		GC–MS LC-MS-QTOF
2-((2-Aminoethyl)-(2-(2-aminoethylamino)-(ethylamino)ethanol	AAEEA*	191		–
2-hydroxyethyl-2-oxazolidone	HEOD	131		GC–MS LC-MS-QTOF
Succinimide	Succ	99		GC–MS
N-Methylsuccinimide	MSucc	113		LC-MS-QTOF
N,N’-Dimethyl-2-imidazolidinone	DMDZ	114		GC–MS
4-[(2-Hydroxyethyl)(nitroso)amino]-1-butanol	HNAP	162		LC-MS-QTOF
1-Nitroso-4-piperidinol	NP	117		LC-MS-QTOF
4-[Butyl(nitroso)amino]-2-butanol	BNAB	173		LC-MS-QTOF
N-(Butyl(nitroso)amino)methyl)acetamide	BNAMA	173		LC-MS-QTOF
(3-Aminopropyl)morpholine	AMM	144		GC–MS
N-(2-hydroxyethyl)-N -methylpiperazine	MPE	144		GC–MS
1,4-Diformylpiperazine	DFP	142		GC–MS
Homoserine	Hom	119		GC–MS
1-Methyl-4-nitrosopiperazine	MNP	129		LC-MS-QTOF
Pyrazole-1-ethanol	PE	112		LC-MS-QTOF
1-Piperazineethanamine	AEP	129		GC–MS
1-(2-(2-Hydroxyethoxy)ethyl) piperazine	HEEP	174		GC–MS LC-MS-Q TOF
N-[2-[3-[N-Aziridyl]propyl]aminoethyl]piperazine	APAP	212		GC–MS
Tetraethylenepentamine	TEP	191		GC–MS

### Amine lose and the concentration of the degradation products

**Fig. 3:**
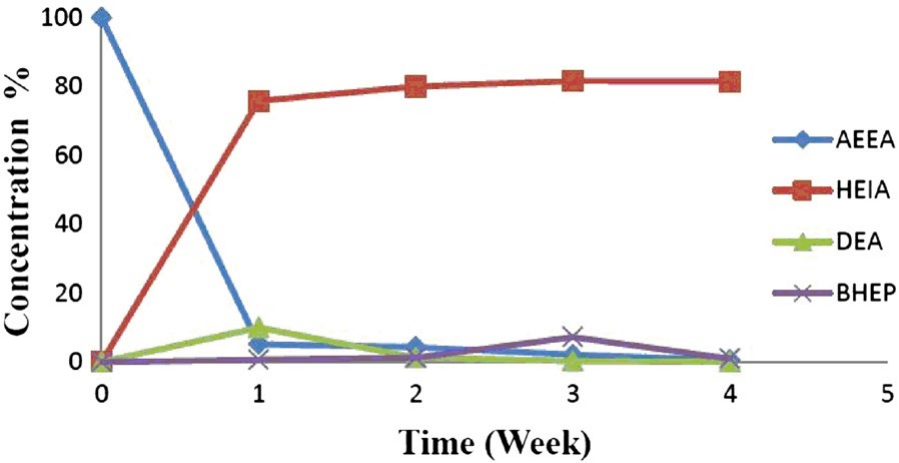
Percentage of AEEA loss and formation of degradation products

The concentration profiles of initial amine (AEEA) and the degradation products of 2-hydroxyethyl-2-oxazolidone (HEIA), (3-(2-Hydroxyethyl)-2-oxazolidinone (HEOD) and 1,4-Bis(2-hydroxyethyl)piperazine (BHEP) concentrations in the samples degraded at 135 °C were obtained as a function of time, as shown in Fig. 3. Concentration of HEIA increased with time and then a little decreased, representing that it is stable product and it plays a role as intermediate after 3 weeks undergoing further reaction rather than a final product. In addition, it is observed that the DEA and BHEP concentrations were very small during the experimental run, which suggests that it may be a key intermediate compound.

### Possible reaction pathway of identified degradation products

An overall reaction pathway has been developed to explain the formation of the major products during the thermal degradation. The objective is to understand the most probable reactions which occur during the thermal degradation process and offer solutions for the elimination of a particular degradation product. The reaction mechanism of the thermal degradation of AEEA was proposed based on the reaction of AEEA with CO_2_ in aqueous solution.

Nevertheless, the proposed reaction mechanism of main degradation products based on this study and literature is debated in this manuscript. Products like HEIA, HEOD, BHEP are the abundant degradation products as per this study. So mechanism postulated in this study is based on the main products. Most of the reaction mechanisms were proposed based on the influence of the ionic species (carbamate and dicarbmate) in the solution.

#### 1-(2-Hydroxyethyl)-2-imidazolidinone (HEIA) and 2-hydroxyethyl-2-oxazolidone (HEOD)

**Scheme 1:**
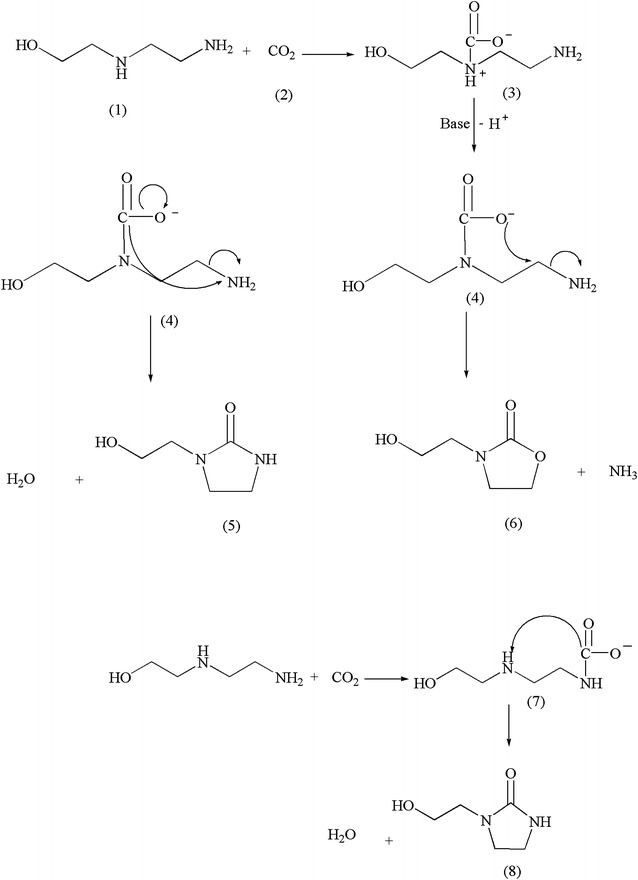
Proposed mechanisms for the formation of HEIA and HEOD

AEEA is a type of diamine compound which contains two nitrogen atoms and reacts with CO_2_ to form several ionic species. It is also known that any ethylenediamine type of structure with two amino groups separated by an ethylene molecule, should form a cyclic urea when exposed to CO_2_ [28]. Cyclic urea, such as 1-(2-Hydroxyethyl)-2 imidazolidinone (HEIA), were observed in MEA degradation and the reaction mechanism in this work is similar to the literature [20, 29]. HEIA is the most abundance degradation products in thermal degradation of AEEA. The formation of HEIA postulated through two different pathways. In Scheme 1, at the presence of CO_2_ HEIA (5) formed at high temperature via the carbamate formation, by dehydration and internal cyclization of the secondary AEEA Carbamate (4). Also There is other possibility of internal cyclization of AEEA secondary carbamate and released of Ammonia to form 2-hydroxyethyl-2-oxazolidone (HEOD) (6). This is analogous to the oxazolidone formation in the presence of ethanolamine and CO_2_, which was described in detail in the thermal degradation of MEA and DEA [20, 28, 30–32]. The other possibility of thermal degradation of carbonated AEEA is generation of HEIA which occurred through internal cyclization of AEEA primary carbamate (7) to generate HEIA (8).

#### 2-Imidazolidinone (HEI)

**Scheme 2:**
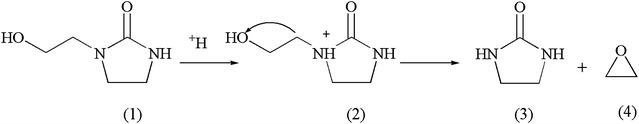
Proposed mechanisms for the formation of HEI

Scheme 2 showed the formation of another type of cyclic urea (HEI) which generated during the thermal degradation of AEEA by the protonation of HEIA (1), followed by the elimination of ethyl alcohol of protonated (HEIA) (2) to produce 2-Imidazolidinone (3) and released ethylene oxide molecule (4).

#### 1,4-Diformylpiperazine (DFP)

**Scheme 3:**
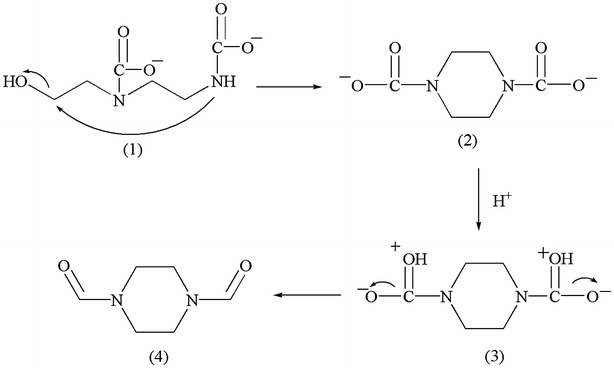
Proposed mechanism for the formation of DFP

Also at high temperature, the ring closure of dicarbamate-AEEA (1) will result in the formation of di-carbamate piperazine (2), followed by the protonation of the carbonyl in both amides (3) which further breaks the O–C bond and forms the π bond to produce the 1,4-Diformylpiperazine (4), according to Scheme 3.

#### 1,4-Bis(2-hydroxyethyl)piperazine (BHEP)

**Scheme 4:**
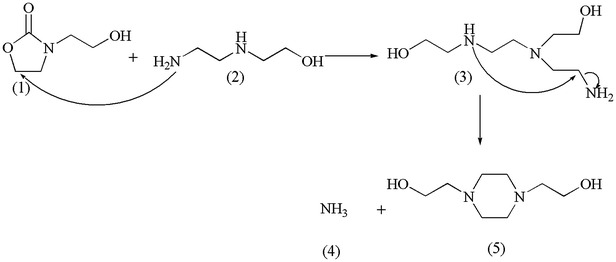
Proposed mechanism for the formation of BHEP

BHEP is a cyclic triamine which is formed by the nucleophilic attack of the HEOD (1) by AEEA (2), where the ring opening promotes the formation of 2-((2-Aminoethyl)-(2-(2-aminoethylamino)-(ethylamino)ethanol (AAEEA) (3). This is followed by the internal cyclization of the AAEEA (3) which releases ammonia (4) and produces the BHEP (5), as shown in Scheme 4.

#### N,N’-Dimethyl-2-imidazolidinone (DMDZ)

**Scheme 5:**
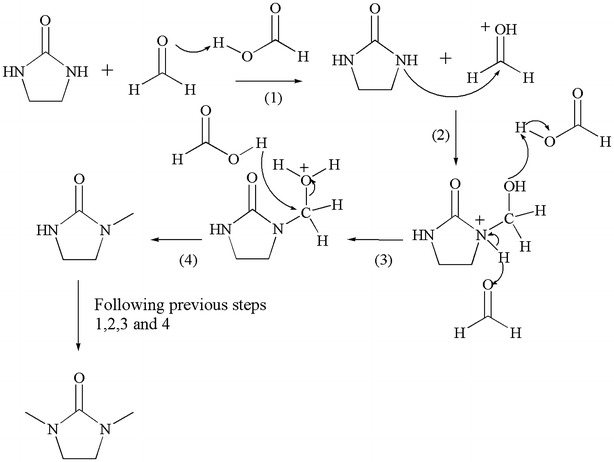
Proposed mechanisms for the formation of DMDZ

The generation of N,N’-Dimethyl-2-imidazolidinone (DMDZ) happen by the attack of formic acid, formaldehyde through a reaction called Eschweiler-Clarke reaction. The reaction began by methylation of amine with protonated formaldehyde to form an iminium ion intermediate, which further react formic acid to form methylated ammonium ion and released CO_2_ as by product. The deprotonation of the ammonium ion affords the final methylated amine product. These steps are repeated twice to give the final tertiary amine product, according to Scheme 5.

### Computational results

**Fig. 4:**
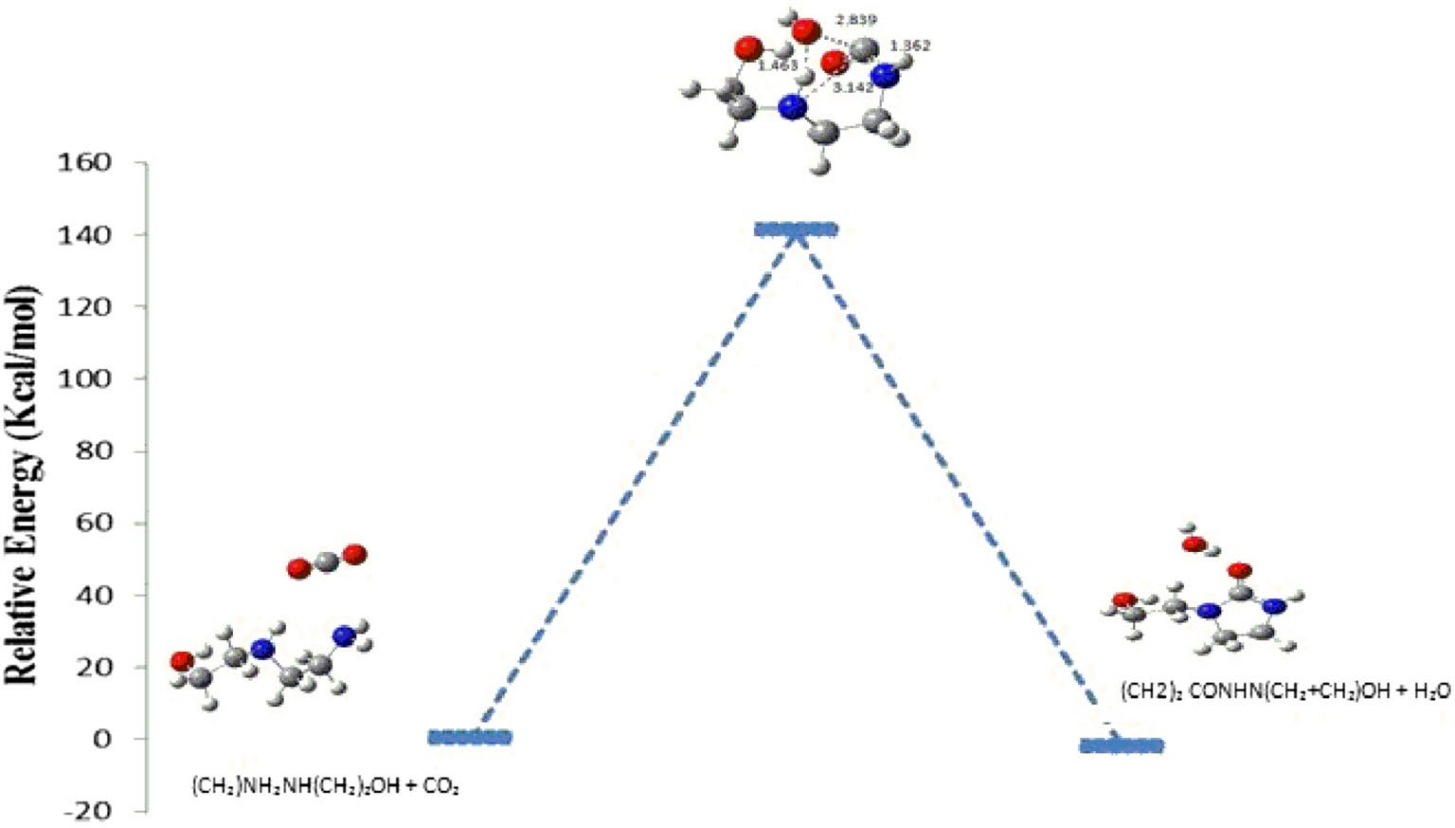
Reaction energy profile for the formation of HEIA (path 1) based on B3LYP/6-311++g(d, p) calculation

**Fig. 5:**
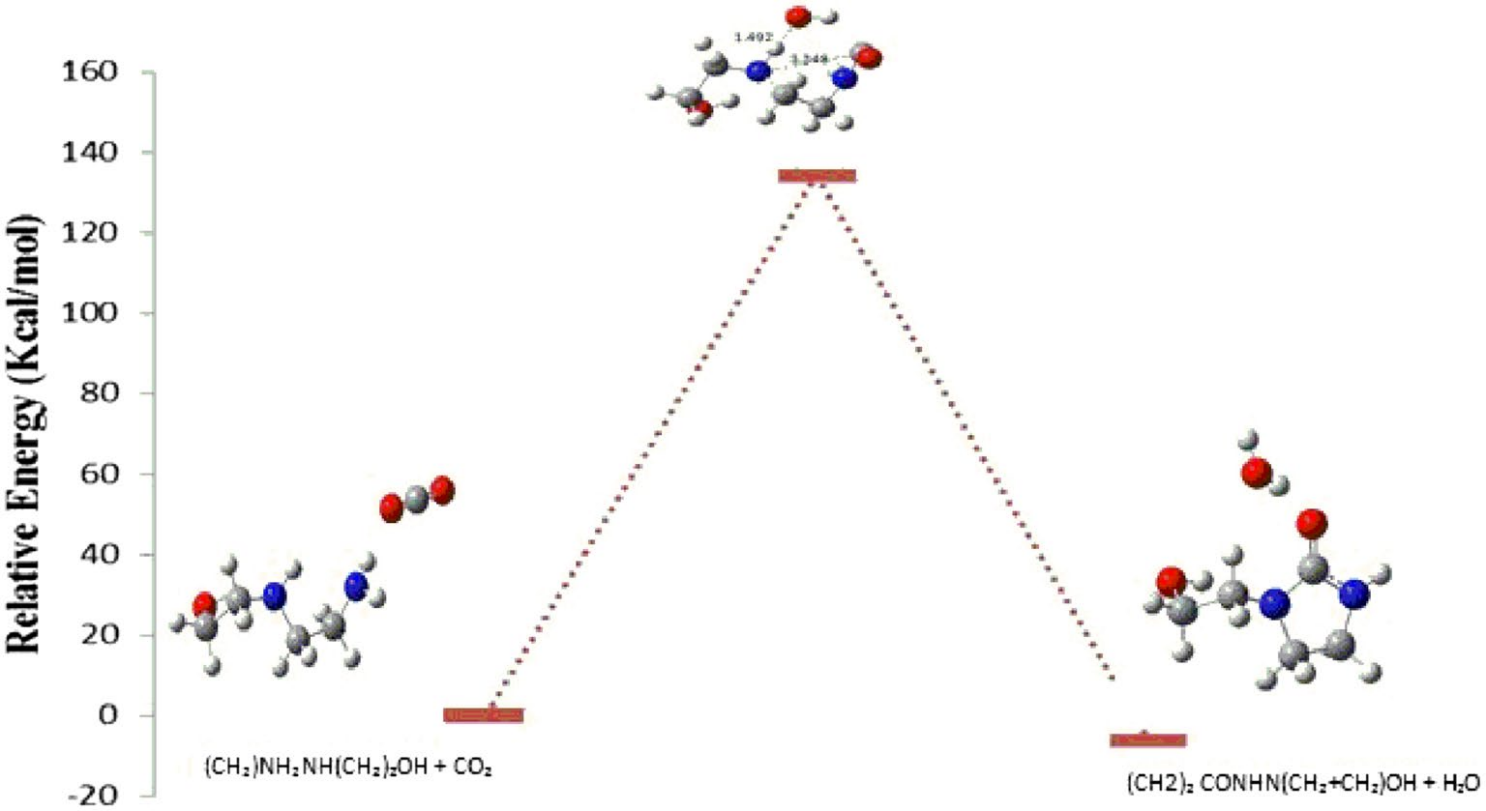
Reaction energy profile for the formation of HEIA (path 2) based on B3LYP/6-311++g(d, p) calculation

In this work, the different pathways of formation of HEIA are investigated theoretically using quantum mechanical calculations. In Figs. 4 and 5, the schematic details of the reaction mechanism, including the transition states, for the formation of HEIA is presented.

The first possibility pathway (path 1) involves the addition of CO_2_ to the secondary amine in AEEA, with simultaneous dehydration to form HEIA and water molecule with an energy barrier of −2.15 kcal/mol. The second mechanism (path 2) involves the addition of CO_2_ to the primary amine in AEEA, with simultaneous dehydration and intermolecular cyclization to form the HEIA and water molecule with an energy barrier of −6.97 kcal/mol. Path 1 has higher activation energy than path 2. The optimized bond lengths in (Å) at the B3LYP/6-31+G(d, p) level of theory are given for the bonds involved in the transition state (Table 4).

**Table 4:** The computed energy and activation barriers for TS1, TS2 obtained at B3LYP/6-311++g(d, p) level of theory for the formation of product according to Figs. 4 and 5

Species	Energy (Ha)	Relative energy (Ha)	Relative energy (kcal/mol)
Reactant 1	−533.1227263	0	0.00
TS1	−532.8979985	0.22472785	141.02
Product 1	−533.1261536	−0.00342728	−2.15
Reactant 2	−533.1150498	0	0.00
TS2	−532.9009546	0.214095141	134.35
Product 2	−533.1261536	−0.011103859	−6.97

## Conclusions

Thermal degradation of 30% AEEA was performed in the presence and absence of CO_2_ loading at 135 °C. AEEA showed high stability in the absence of CO_2_, and no degradation products were identified. However, AEEA degraded significantly in the presence of CO_2_ and twenty-seven degradation products were identified by GC–MS based on the (NIST) library search, and based on LC-QTOF-MS search. 2-hydroxyethyl imidazolidone (HEIA) was the most abundant degradation product, which contributed to the loss of the AEEA concentration. The reaction energy of HEIA formation were calculated for the both pathways of its formations and found to be −2.15 kcal/mol and −6.97 kcal/mol. Degradation rates of AEEA show that it may not be a choice of commercialization or large CO_2_. However, under lab scale more investigation may be conducted by using degradation inhibitors. Or another way may be modification of AEEA by addition of an alkyl group to the amines groups could be a possible way to prevent the carbamate formation.
